# The Dual Dose-Dependent Effects of Corticosterone on Hippocampal Cell Apoptosis After Traumatic Brain Injury Depend on the Activation Ratio of Mineralocorticoid Receptors to Glucocorticoid Receptors

**DOI:** 10.3389/fphar.2021.713715

**Published:** 2021-07-26

**Authors:** Bin Zhang, Mengshi Yang, Qiongyu Yan, Xiaojian Xu, Fei Niu, Jinqian Dong, Yuan Zhuang, Shenghua Lu, Qianqian Ge, Baiyun Liu

**Affiliations:** ^1^ Department of Neurosurgery, Beijing Tiantan Hospital, Capital Medical University, Beijing, China; ^2^ Department of Pharmacy, Beijing Tiantan Hospital, Capital Medical University, Beijing, China; ^3^ Beijing Neurosurgical Institute, Capital Medical University, Beijing, China; ^4^ Department of Neurosurgery and Beijing Key Laboratory of Central Nervous System Injury, Beijing Tiantan Hospital and Beijing Neurosurgical Institute, Capital Medical University, Beijing, China; ^5^ Nerve Injury and Repair Center of Beijing Institute for Brain Disorders, Beijing, China; ^6^ China National Clinical Research Center for Neurological Diseases, Beijing, China

**Keywords:** traumatic brain injury, corticosterone, mineralocorticoid receptor, glucocorticoid receptor, apoptosis

## Abstract

In our recent studies, we reported that mineralocorticoid receptor (MR) had the opposite effects of glucocorticoid receptor (GR) on neural cell survival after traumatic brain injury (TBI). However, whether short-term use of high-dose natural glucocorticoids, which are mixed agonists of both MR and GR, leads to neurotoxic effects by inducing excessive GR activation is unclear, as is the threshold GR activation level and the possible signaling pathways remain unclear. In this study, we examined the dual dose-dependent effects of corticosterone (CORT) on spatial memory, hippocampal cell survival and receptor-mediated downstream signaling pathways after TBI. We found that different doses of CORT exhibited dual effects on hippocampal cell survival and rat spatial memory. Low doses of CORT (0.3 and 3 mg/kg) significantly increased MR activation, upregulated Akt/CREB/Bad phosphorylation and Bcl-2 concentration, reduced the number of apoptotic neural cells, and subsequently improved rat spatial memory. In contrast, a high dose of CORT (30 mg/kg) exerted the opposite effects by overactivating GR, upregulating P53/Bax levels, and inhibiting Erk/CREB activity. The results suggest that the neuroprotective and neurotoxic effects of endogenous GC depend on a threshold level and that a higher dose of GC, even for short-term use, should be avoided after TBI.

## Introduction

Since their discovery in the 1950s, glucocorticoids (GCs), especially synthetic GCs, have been widely used to treat a wide range of diseases. However, their clinical use is limited by severe adverse effects, such as diabetes mellitus, osteoporosis, hypertension, and increased risk of infection (Rhen et al., 2005; Cain et al., 2017; Vandewalle et al., 2018). In recent years, the central nervous system (CNS) side effects of synthetic GCs, namely, cognitive, mental, and stress dysfunction, have caused serious concern (Judd et al., 2014; Laugesen et al., 2021). In the CNS, the effects of GCs are mediated by two receptors: the mineralocorticoid receptor (MR) and glucocorticoid receptor (GR) (Reul et al., 1985; Sarabdjitsingh et al., 2009). Endogenous GCs (primarily cortisol in humans and corticosterone (CORT) in rodents) are mixed agonists of both MR and GR but have a 10-fold higher affinity for MR, while synthetic GCs (for example, methylprednisolone (MP) and dexamethasone (DEX)), with higher affinity for GR than for MR, are more potent anti-inflammatory and immunosuppressive agents (de Kloet et al., 2018). Experimental studies have revealed that sufficient activation of MR is crucial for the normal functions of many structures in the brain, including the hippocampus and hypothalamus and that excessive or prolonged activation of GR caused by long-term synthetic GR agonist treatment or excessive endogenous GCs secretion (for example, due to chronic stress or Cushing syndrome) causes severe cognitive, stress, and mood disorders (Krugers et al., 2010; Fardet et al., 2012; de Kloet et al., 2014, 2018; Burkhardt et al., 2015).

Secondary brain injury, including brain edema, increased intracranial pressure, hemorrhage/ischemia, apoptosis, inflammation, excitotoxicity, oxidative stress, calcium dysregulation, and axonal degeneration, can occur after primary brain injury (Maas et al., 2017; Morganti-Kossmann et al., 2019). Apoptosis is a major form of cell loss after traumatic brain injury (TBI), leading to severe neurological and psychiatric complications that seriously influence the quality of life of survivors or even threaten their lives (Zhang et al., 2005). Synthetic GCs have been commonly used in patients with moderate and severe TBI to alleviate secondary brain injury. However, the outcomes of previous clinical trials were inconsistent, with some trials showing opposite results (Cooper et al., 1979; Braakman et al., 1983). One study reported detrimental effects of MP on TBI patients (Roberts et al., 2004). Our recent findings showed that the deleterious effects of high-dose GCs might be central origin. We found that excessive activation of GR by MP or DEX increased neuronal apoptosis by activating GR in the hippocampus and hypothalamus, exacerbated spatial memory deficits and stress dysfunction, and increased mortality. In contrast, low-dose corticosterone (CORT) replacement restored plasma CORT levels, refilled MRs, and promoted neuronal survival, but the dose-dependent effects of CORT and the receptor-mediated downstream mechanisms remain unclear (Zhang et al., 2020a). Other researchers have shown that cAMP-responsive element-binding protein (CREB) and several intracellular pathways are crucial for the regulation of apoptosis-related proteins and neuronal apoptosis after TBI. (Zuo et al., 2016; Wu et al., 2019). However, whether short-term use of high-dose CORT, a mixed agonist of both MR and GR, has deleterious effects on hippocampal cells, the dose of CORT that activates GR, and the components of apoptosis-related signaling pathways that engage in cross talk with CORT in the hippocampus after TBI remain unclear.

In this study, we hypothesize that different doses of CORT play opposite roles in hippocampal cell survival and spatial memory maintenance after TBI. Low doses of CORT restore the activation of MR and promote the survival of hippocampal cells, whereas high doses of CORT increase the neural cell apoptosis rate by activating GR. CREB and Bcl-2 family proteins might be involved in the dose-dependent effects of CORT. By using a rat TBI model and different CORT doses, we tested our hypothesis and found a CORT dose that fully activates MR but does not overactivate GR.

## Materials and Methods

### Animal Preparation and Controlled Cortical Impact (CCI)

Adult male Sprague–Dawley rats (weighing 300–320 g) were used in the present study. The rats were housed individually under controlled conditions (temperature, 22 ± 1°C; humidity, 60%) with a 12-h light/dark cycle for 7 days before surgery. Food and water were available ad libitum. All experimental procedures were approved by the Capital Medical University Institutional Animal Care and Use Committee (approval number: 201802001).

CCI was performed as previously described (Zhang et al., 2020a; Zhang et al., 2020b; Zhang et al., 2020c). Briefly, all rats were anesthetized by isoflurane inhalation, and the head was fixed in a stereotaxic frame. A 6-mm craniotomy was made in the middle of the right parietal bone, leaving the underlying dura intact. Then, the rats were subjected to impact with parameters we previously described (moderate TBI model: velocity, 2.8 m/s; compression time, 85 ms; depth, 2 mm; and diameter of impactor tip, 5 mm). Sham-operated rats underwent the same procedure without percussion. The body temperature was maintained at 37.0 ± 0.5°C with a thermal pad throughout the surgery.

### Experimental Groups and Corticosterone Treatments

All 168 rats (*n* = 24 per group) were assigned on the basis of CCI and treatment into the following groups: 1) a sham control group (sham); 2) CCI group (CCI); 3) CCI + CORT (0.3, 3, and 30 mg/kg, ab143597, Abcam) group (CCI + CORT1, CCI + CORT2, and CCI + CORT3, respectively); 4) CCI + spironolactone (SPIRO) (50 mg/kg, ab141289, Abcam) + CORT (0.3 mg/kg) group (CORT1+SPIRO); and 4) CCI + mifepristone (50 mg/kg, ab141289, Abcam) + CORT (30 mg/kg) group (CORT3+RU486). All drugs were dissolved in sterile 0.9% NaCl solution containing dimethyl sulfoxide (DMSO, at a final concentration <1%, Sigma). Rats in the sham and CCI groups received an equal volume of solvent. Drugs were administered intraperitoneally for 3 days after CCI. To effectively block MR and GR function, SPIRO and RU486 (administered twice per day) were given for 2 days before CCI and 3 days after CCI. All drug doses were chosen based on pilot experiments performed in our laboratory and in our previous study (Zhang et al., 2020a).

Morris water maze (MWM) (*n* = 24 per group) assessments were repeatedly performed in all groups after TBI. The rats were euthanized by decapitation on days 3 after injury. Brains (*n* = 8 per group) processed for hematoxylin and eosin (H and E) staining, the terminal deoxynucleotidyl transferase deoxyuridine triphosphate (dUTP) nick end labeling (TUNEL) assay, and Immunofluorescence staining were removed and fixed in 4% paraformaldehyde for 24 h. After fixation, they were embedded in paraffin, processed into 5 μm-thick coronal paraffin sections at the level of the hippocampus (2.8–3.8 mm posterior to bregma) were made according to the Paxinos atlas of the rat brain (Paxinos et al., 1986), and subsequently affixed to poly-l-lysine-coated slides. Brains (*n* = 16) processed for Western blot (*n* = 8 per group for total protein; *n* = 8 per group for nuclear protein) were rapidly removed, and the right hippocampus was dissected at 4°C, frozen in liquid nitrogen, and then stored at −80°C before further processing.

### Morris Water Maze

The MWM test was used to test spatial memory in this study. Rats (*n* = 24 per group) were trained for the MWM test before injury according to a protocol previously described (Zhang et al., 2020a). Each rat underwent four trials per day for five consecutive days (8–4 days before CCI), during which they were to find the platform submerged below the water (20 ± 2°C, with nontoxic black ink). The pool (150 cm in diameter) was divided into four equal quadrants (northwest, northeast, southwest, and southeast), and a target platform (10 cm in diameter) was hidden 2 cm below the surface of water in the middle of the southwest quadrant. Each trial (with a 5-min interval between tests) was initiated from different positions (north, east, southeast, and northwest) and lasted no longer than 120 s. If the animal reached the platform within 120 s, the time was recorded as the latency time. If the rat failed to find the platform, the trial was terminated, and the animal was placed on the platform for 15 s. The latency time was recorded as 120 s. To assess spatial memory, a probe trial was conducted 3 days before and after CCI. The hidden platform was removed, and the rats started the test at the northeast position, 180° from the original hidden platform position. The percentage of time spent and distance traveled in the goal quadrant during the 30-s (total) swimming period was recorded.

### Nick End Labeling Assay and H and E Staining

To assess the survival and apoptosis of hippocampal cells, H and E staining and TUNEL assays (Roche, Germany) were carried out as we previously described (Zhang et al., 2020a). Briefly, after rehydration, the tissue slides were incubated in hematoxylin (ab245880, Abcam) for 5 min, rinsed two times with water and then dipped in blue reagent for 10 s. After two rinses with distilled water, the slides were dipped in ethanol and then incubated in eosin solution for 2 min (ab245880, Abcam) and then rinsed with distilled water and ethanol. For the TUNEL assay, tissue sections were incubated with a proteinase K working solution (20 mg/ml proteinase K in 10 mM Tris-HCl buffer, pH 7.5–8.0) for 10 min at 37°C. The sections were rinsed twice with 0.01 M phosphate-buffered saline (PBS) (pH 7.4) and subsequently incubated at 37°C with the TUNEL reaction mixture for 1 h. Finally, 3,3-diaminobenzidine (DAB) substrate was added to the mixture, and the color was allowed to develop for 10 min. The sections were lightly counterstained with hematoxylin and mounted with neutral balsam. A MIDI FL (3DHISTECH, Hungary) system was used to obtain digital images of the brain sections. A digital image analysis system (3DHISTECH, Hungary) was used to count the number of surviving and apoptotic cells in the ipsilateral hippocampus (in three sections) as previously described (Zhang et al., 2020a). In addition, the number of cells in the three tissue sections of the ipsilateral hippocampus were counted (at 50-µm intervals). TUNEL-positive cells presenting morphological changes characteristic of apoptosis were also counted in the ipsilateral hippocampus magnified at ×200. The mean number of apoptotic cells is presented as the number of apoptotic cells in each sample. To determine the cell numbers in the ipsilateral hippocampus, three nonoverlapping zones in the CA3 and CA1 ipsilateral areas and nine zones in the ipsilateral dentate gyrus were evaluated. These data are presented as the number of hippocampal cells per mm and the total number of TUNEL-positive cells. All analyses were performed in a blinded fashion.

### Immunofluorescence Staining

Immunofluorescence staining for cleaved caspase-3 was performed as we previously described (Zhang et al., 2020c). 5 μm-thick coronal paraffin sections adjacent to those used for TUNEL staining were assessed. Briefly, the brain sections were incubated in 3% hydrogen peroxide for 0.5 h and then blocked with normal bovine serum for 0.5 h. The brain sections were washed three times and incubated overnight with rabbit polyclonal anti-cleaved caspase-3 (1:400, CST, #9664) at 4°C. The slices were then washed with PBS and incubated with Alexa Fluor 647–conjugated donkey anti-rabbit IgG at room temperature for 2 h. Finally, the samples were counterstained with 4′,6-diamidino-2-phenylindole (DAPI) (Sigma-Aldrich, St. Louis, MO) for 10 min. Digital images of whole-brain sections were obtained with a MIDI FL system (3DHISTECH, Hungary).

### Western Blot Analysis

In this study, we tested levels of the phosphorylated proteins to evaluate their real activities after TBI. Total protein (*n* = 8 per group) and nuclear protein (*n* = 8 per group) were extracted as previously described (Tao et al., 2015). An equal amount of each protein sample (30 μg) was separated by SDS–PAGE, and then, the separated proteins were transferred onto polyvinylidene fluoride membranes. The membranes were incubated with 5% nonfat milk or BSA for 2 h and then allowed to react overnight at 4°C with primary antibody rabbit polyclonal against phosphorylated-Akt (*p*-Akt, ser 473, 1:5,000; ab81283), rabbit polyclonal against *p*-Erk (1:1,000, CST, #4370), rabbit polyclonal against -CREB (ser133, 1:1,000, ab32096), rabbit polyclonal against P53 (1:1,000, ab131442), rabbit polyclonal against Bax (1:1,000, ab32503), rabbit polyclonal against p-Bad (S136, 1:1,000, ab28824), rabbit polyclonal against cleaved caspase-3 (1:1,000, #9664, CST), rabbit polyclonal against Bcl-2 (1:1,000; ab196495), mouse monoclonal against MR (1:400, ab2774), rabbit polyclonal against GR (1:200, ab3578), rabbit monoclonal against β-Actin (1:5000, ab179467, Abcam, United Kingdom), and rabbit polyclonal against histone H3 (1:400, Millipore Co.). Next, the membranes were incubated with secondary antibodies for 2 h at room temperature. The blots were visualized using chemiluminescence (Bio Spectrum 500 Imaging System; UVP Co., Upland, CA, United States). The relative band density was measured with ImageJ software (version 1.49) and normalized to that of β-actin. The level of each protein compared to that of the sham controls was calculated as a percentage of each sample.

### Co-Immunoprecipitation Assays

Co-immunoprecipitation (co-IP) assays were performed to assess the protein-protein interactions between Bcl-2, Bad, and Bax. According to previously described methods (Liang et al., 2021), a 500-μg sample of total protein was first pretreated with either rabbit polyclonal anti-Bcl-2 (1:1,000; ab196495, Abcam, United Kingdom) or rabbit polyclonal anti-Bad (1 μg/ml, ab90435, Abcam). A total of 20 μL of protein A/G agarose (Sigma) was added to each sample, and the mixtures were incubated overnight at 4°C and then centrifuged for 1 min at 12,000 *g*. To remove nonspecifically bound proteins, the precipitates were rinsed four times with NP-40 buffer. Agarose-bound immunocomplexes were then released by resuspension in loading buffer containing a denaturing agent. IgG was used as a negative control for precipitation. The protein levels of Bcl-2, Bax, and Bad in the precipitates were then assessed by western blotting as described above and probed with antibodies against Bcl-2, Bax, and Bad.

### Statistical Analysis

All the data are depicted as the means ± SD. The data were analyzed using SPSS 22.0 software (IBM Corporation, United States). The time spent and distance travelled in the target quadrant recorded as a percentage, the number of TUNEL-positive cells, neural cell counts, and western blot data were statistically analyzed using one-way analysis of variance (ANOVA) followed by Tukey’s post hoc test, and the *p* value was adjusted by Bonferroni correction. Repeated measures ANOVAs were performed to analyze the escape latency of rats in the MWM test. A *p*-value < 0.05 was considered statistically significant.

## Results

### Dose-dependent Effects of Corticosterone on Rat Spatial Memory After Traumatic Brain Injury

**FIGURE 1:**
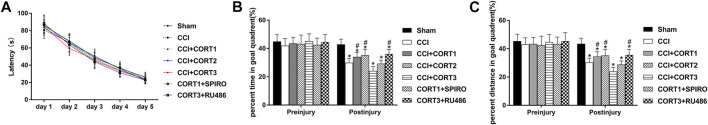
Dose-dependent effects of COR*T treatments on spatial memory after CCI. **(A)** Quantification of the escape latency of rats during the MWM test before CCI. **(B, C)** The percentage of time spent and distance travelled in the goal quadrant during the probe trial before and after CCI (*n* = 24 per group). ^*^ *p* < 0.05 versus the sham control group; ^#^ *p* < 0.05 versus the CCI control group. The data are presented as the means ± SDs.

To assess the baseline spatial learning and memory ability of the rats, latency time was determined through probe trials performed before TBI. Our recent study showed that mild motor function disturbance occurred immediately after CCI, but was improved rapidly within 24 h, and the motor test showed no difference among rats in all experimental groups (Zhang et al., 2020b). The escape latencies for all the experimental groups were significantly reduced (*p* < 0.05) from 1 day to 5 days, but no significant differences were observed between the different groups at each time point [repeated ANOVAs, F (4, 644) = 8,365.233 for time, F (6, 161) = 0.866, *p* > 0.05 for treatment] (Figure 1A). In this study, we used probe trials to evaluate the dose-dependent effects of CORT on rat spatial memory. No significant difference in the percent time and distance between any groups before TBI [one-way ANOVA, F(6, 167) = 1.289, *p* > 0.05, for percent time; F (6,167) = 1.181, *p* > 0.05, for percent distance], but this percentage was significantly reduced after TBI. Rats that received lower doses of CORT (0.3 and 3 mg/kg) exhibited improved recovery of spatial memory and spent a significantly greater percentage of time and distance in the goal quadrant than the rats the CCI control group. In contrast, a high dose of CORT (30 mg/kg) significantly reduced the percentage of time and distance in the goal quadrant, compared with the rats in the CCI group. [one-way ANOVA, F (6, 167) = 129.755, *p* < 0.05 for percent time; F (6,167) = 75.033, *p* < 0.05 for percent distance] (Figures 1B,C). The opposite effects of low-dose and high-dose CORT were counteracted by SPIRO and RU486, respectively.

### Dose-dependent Effects of Corticosterone on the Survival of Hippocampal Cells After Traumatic Brain Injury

**FIGURE 2:**
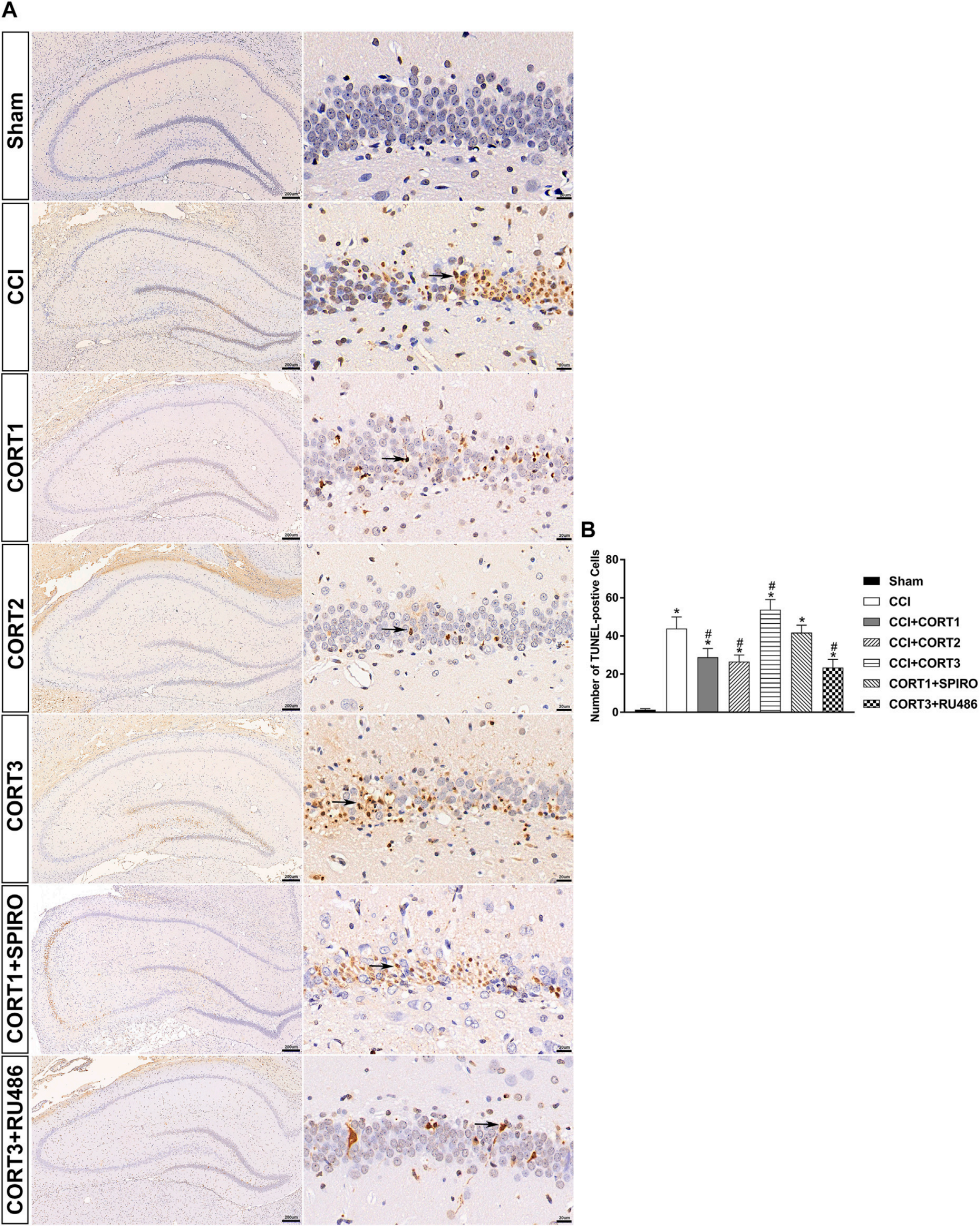
Dose-dependent effects of CORT treatments on the apoptosis of ipsilateral hippocampal neurons after CCI. **(A)** Representative images of TUNEL-positive cells (black arrow) in the ipsilateral hippocampus 3 days after CCI. **(B)** Quantification of apoptotic neurons in the ipsilateral hippocampus. *p* < 0.05 versus the sham control group; ^#^ *p* < 0.05 versus the CCI control group. The data are presented as the means ± SD. Scale bar = 200 μM for the left column of Panel 2A and 20 μM for the right column of Panel 2A.

**FIGURE 3:**
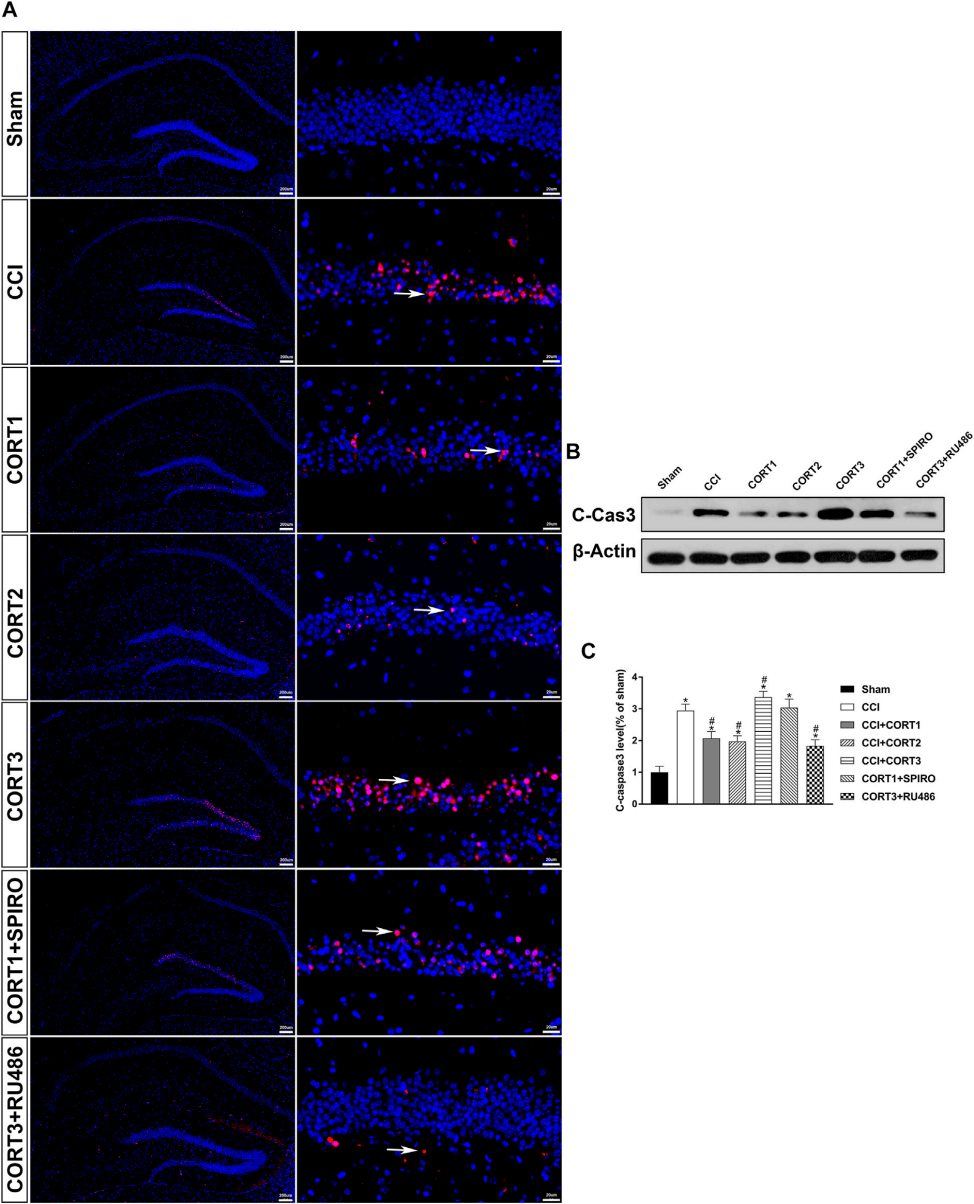
Dose-dependent effects of CORT treatments on the level of cleaved caspase-3 after CCI. **(A)** Representative images of cleaved caspase-3 (red, white arrow) immunofluorescence in the ipsilateral hippocampus. **(B)** Representative western blot images of cleaved caspase-3. **(C)** Quantification of cleaved caspase-3 expression. The relative band density was measured with ImageJ software (version 1.49) and normalized to that of β-actin, and the concentration percentage compared to that of sham controls was calculated for each sample. *p < 0.05 compared to the sham group; #*p*˂0.05 compared to the CCI control group. The data are presented as the means ± SDs of eight animals per group. Scale bar = 200 μM for the left column of Panel 3A and 20 μM for the right column of Panel 3A.

**FIGURE 4:**
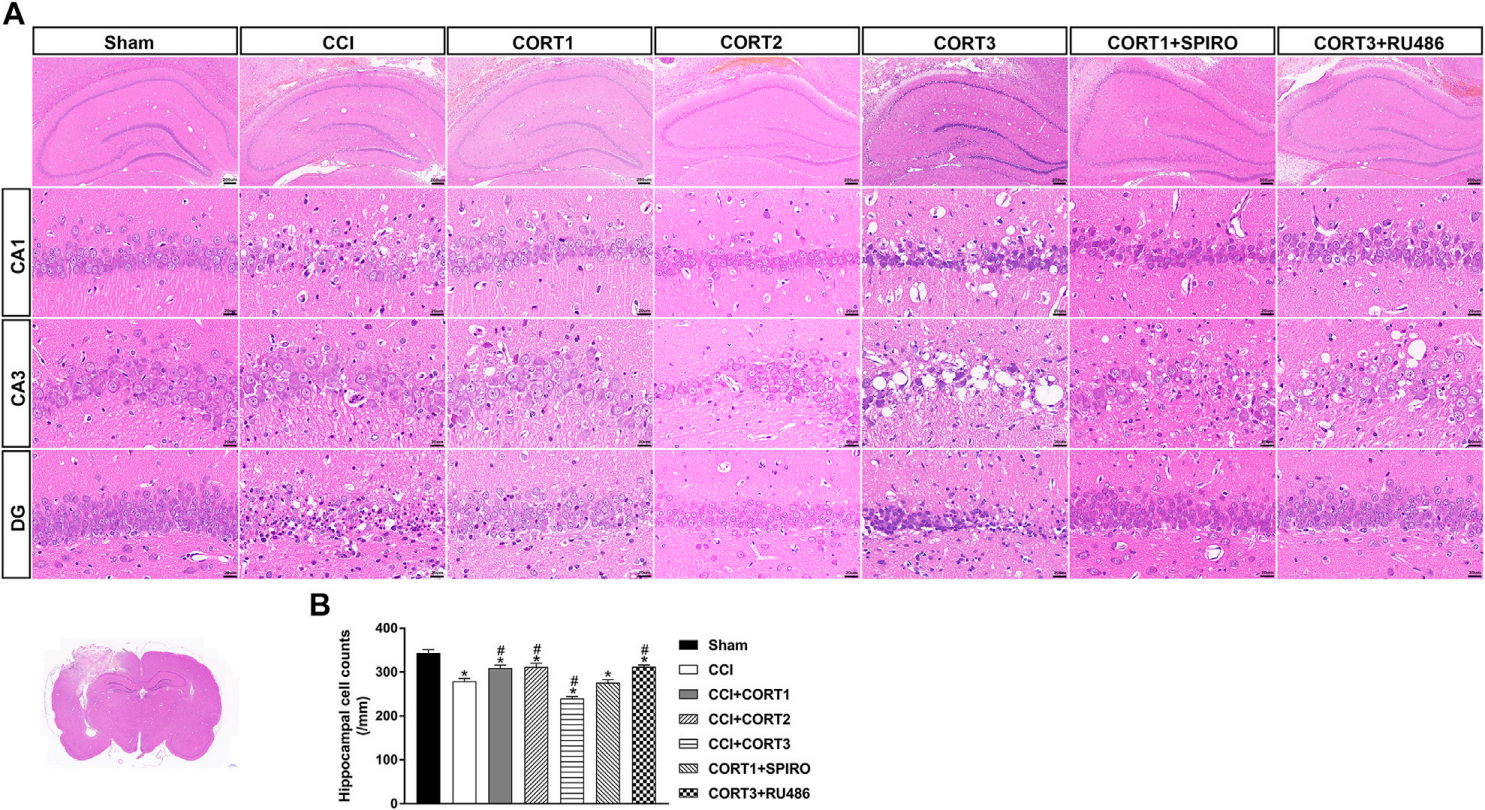
Dose-dependent effects of CORT treatments on neural cell in the ipsilateral hippocampus after CCI. **(A)** Representative images of H and E staining samples showing neural cells in ipsilateral hippocampal subareas. **(B)** Quantification of cells in the ipsilateral hippocampus. ^*^ *p* < 0.05 versus the sham control group; ^#^ *p* < 0.05 versus the CCI control group. The data are presented as the means ± SDs. Scale bar = 200 μM for the upper row of Panel 4A and 20 μM for CA1, CA3, and DG rows of Panel 4A.

In accordance with previous studies, our recent studies revealed that secondary injury, including inflammation, edema, and apoptosis, peaked approximately 48–72 h after TBI. In the present study, we tested cell survival and apoptosis in the ipsilateral hippocampus on postinjury day 3 (Figures 2A, 3A, 4A). Our results showed that the number of TUNEL-positive cells and the level of cleaved caspase-3 were significantly increased (*p* < 0.05) (Figures 2B, 3B) and that the number of surviving cells was significantly reduced (*p* < 0.05) in the ipsilateral hippocampus (Figure 4B). Lower doses of CORT (0.3 and 3 mg/kg) significantly reduced the level of cleaved caspase-3 and the number of apoptotic cells (*p* < 0.05) and subsequently increased the number of surviving cells compared with that in the CCI control rats (*p* < 0.05). In contrast, high-dose CORT (30 mg/kg) significantly increased the level of cleaved caspase-3 and the number of TUNEL-positive cells and concomitantly reduced the number of neural cells (*p* < 0.05). In addition, the dose-dependent effects of CORT on cell survival were counteracted by SPIRO and RU486 pretreatment [one-way ANOVA, F(6, 55) = 124.749 for apoptosis; F(6, 55) = 271.516 for cell counts, *p* < 0.05; F(6, 55) = 130.236 for cleaved caspase-3].

### Dose-dependent Effects on Cell Survival Were Mediated by Two Receptor Systems: Mineralocorticoid Receptors and Glucocorticoid Receptors

**FIGURE 5:**
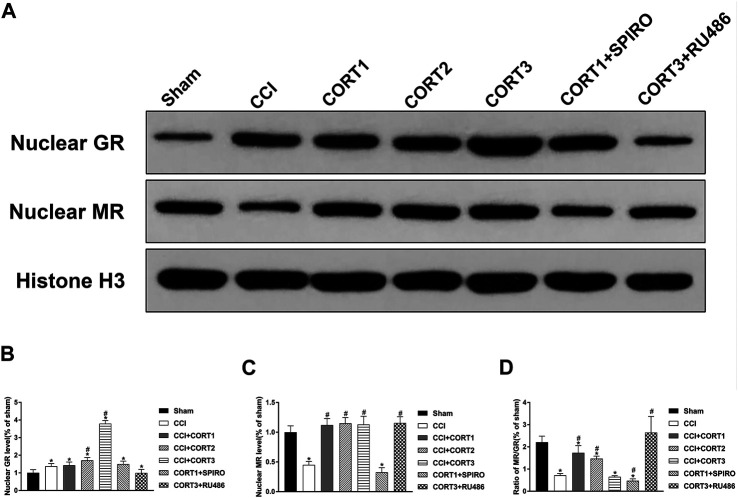
Dose-dependent effects of CORT treatments on the activation of MR and GR. **(A)** Representative western blot images of nuclear MR and GR. **(B–D)** Quantification of nuclear MR, nuclear GR, and the ratio of MR/GR. The relative band density was measured with ImageJ software (version 1.49) and normalized to that of histone H3, and the concentration percentage compared to that of sham controls was calculated for each sample. *p < 0.05 compared to the sham group; #*p*˂0.05 compared to the CCI control group. The data are presented as the means ± SDs of eight animals per group.

To assess the MR and GR activation level, the nuclear translocation levels of MR and GR were examined in this study (Figure 5A). We found that nuclear MR and the ratio of MR/GR were significantly (*p* < 0.05) reduced in the ipsilateral hippocampus after TBI. CORT treatments significantly increased nuclear MR compared with that of the TBI control group (*p* < 0.05), but only low doses of CORT (0.3 and 3 mg/kg) significantly increased the MR/GR ratio compared with that of the CCI group. When the dose of CORT was increased to 3 mg/kg, the nuclear GR level increased, peaking at 30 mg/kg (*p* < 0.05). However, a significantly reduced MR/GR ratio compared to that of the CORT1 group was found only in the high-dose CORT group (*p* < 0.05). SPIRO and RU486 pretreatment inhibited the increase in MR and GR activation, respectively [one-way ANOVA, F (6, 55) = 96.631 for MR; F (6, 55) = 238.763 for GR; and F (6, 55) = 53.239 for MR/GR] (Figure 5B).

### Dose-dependent Effects of Corticosterone on the Concentrations and Interactions of Bcl-2 Family Proteins

**FIGURE 6:**
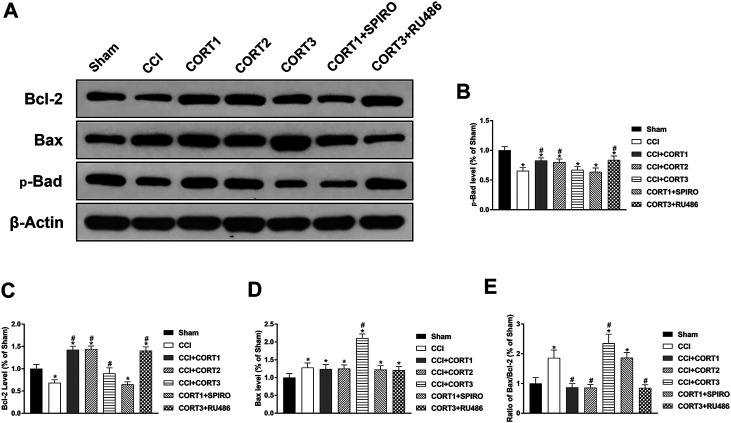
Dose-dependent effects of CORT treatments on the activation and concentration of Bcl-2 family proteins. **(A)** Representative western blot images of Bcl-2, Bax and p-Bad expression. **(B–E)** Quantification of p-Bad, Bcl-2, Bax and Bax/Bcl-2. *p < 0.05 versus the sham control group. The relative band density was measured with ImageJ software (version 1.49) and normalized to that of β-actin, and the concentration percentage compared to that of the sham controls was calculated for each sample. *p < 0.05 compared to the sham group; #*p* < 0.05 versus the CCI control group. The data are presented as the mean ± SD of eight animals in each group.

Both extrinsic and intrinsic forms of apoptosis are regulated by Bcl-2 family proteins, which include antiapoptotic (Bcl-2) and proapoptotic (Bax and Bad) proteins. The ratio of Bax/Bcl-2 and the interactions between these proteins are important factors in determining whether apoptosis occurs. Figure 6A shows the levels of Bcl-2, Bax, and p-Bad. The levels of p-Bad and Bcl-2 in the ipsilateral hippocampus was significantly (*p* < 0.05) reduced 3 days after TBI (Figures 6B,C), whereas the expression of Bax and the ratio of Bax/Bcl-2 were significantly (*p* < 0.05) increased (Figures 6D,E). Treatment with low doses of CORT (0.3 and 3 mg/kg) significantly increased Bcl-2 and p-Bad and reduced the ratio of Bax/Bcl-2 compared with CCI treatment alone (*p* < 0.05). In contrast, high-dose CORT treatment significantly reduced the level of p-Bad and increased the ratio of Bax/Bcl-2 (*p* < 0.05) by upregulating Bax level [one-way ANOVA, F(6, 55) = 135.379, *p* < 0.01 for Bcl-2; F(6, 55) = 77.776, *p* < 0.01 for Bax; F(6, 55) = 41.326, *p* < 0.01 for p-Bad; and F(6, 55) = 81.04, *p* < 0.01 for Bax/Bcl-2]. The dose-dependent effects of CORT on Bcl-2 family protein were counteracted by RU486 and SPIRO pretreatment, which demonstrated that these effects were GR- and MR-mediated.

**FIGURE 7:**
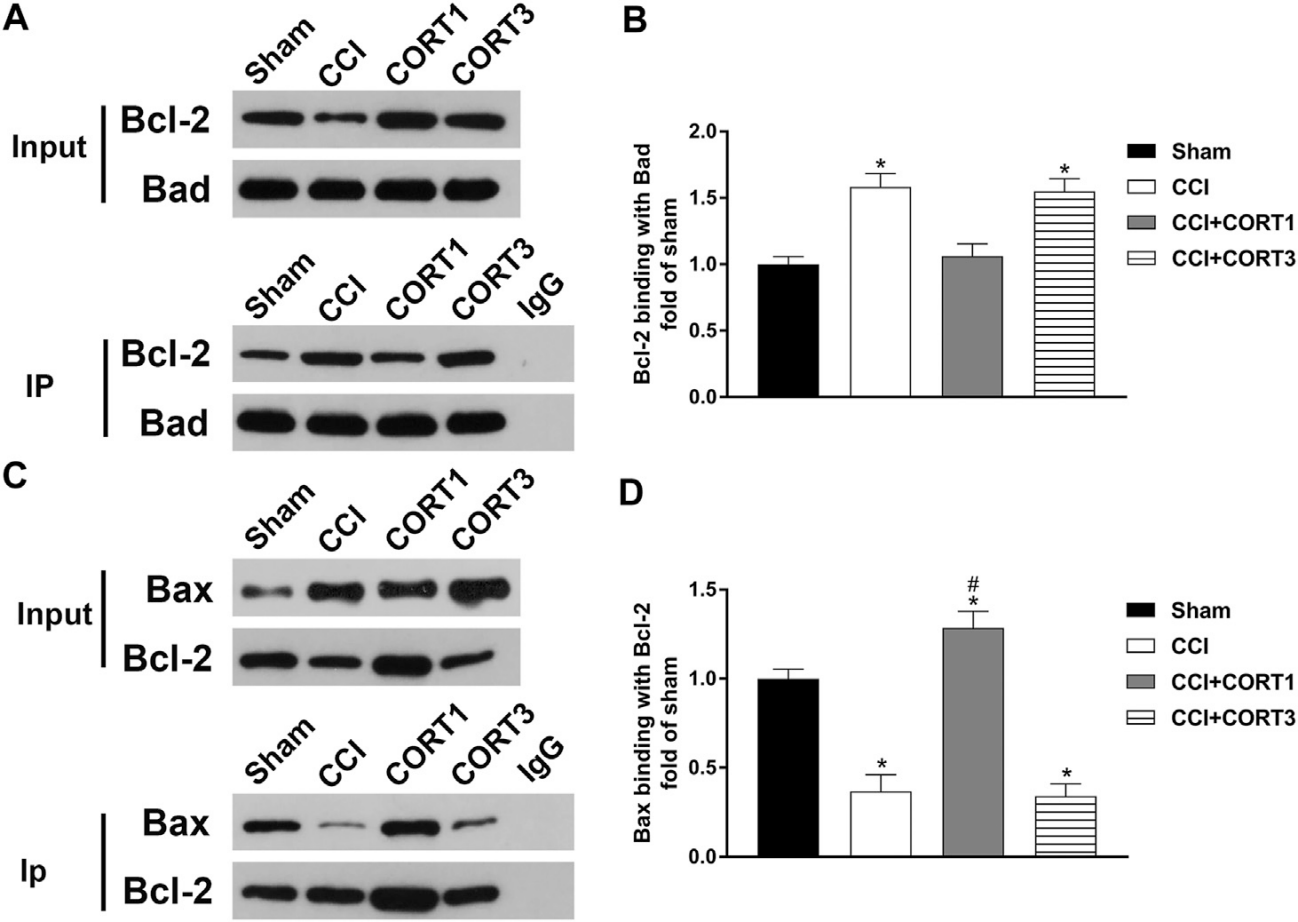
Dose-dependent effects of CORT treatments on the interactions between Bcl-2 family proteins. **(A, B)** Representative western blot images and quantification of Bcl-2 levels (binding with Bad) as assessed by co-IP with a Bad antibody. **(C, D)** Representative western blot images and quantification of Bax levels (binding with Bcl-2) as assessed by co-IP with a Bcl-2 antibody. The concentration percentage compared to that of sham controls was calculated for each sample. *p < 0.05 compared to the sham group; #*p* < 0.05 versus the CCI control group. The data are presented as the mean ± SD of eight animals in each group.

In addition, the interactions between Bad, Bcl-2 and Bax directly reflected their activity levels. Bad can combine with Bcl-2 to inhibit the activity of Bcl-2, which exhibits an antiapoptotic effect by binding to Bax. The co-IP results showed that the level of Bcl-2 combined with Bad was significantly increased (*p* < 0.05), and the level of Bax combined with Bcl-2 was significantly reduced (*p* < 0.05) on postinjury day 3. A low dose of CORT (0.3) significantly inhibited (*p* < 0.05) the interaction between Bad and Bcl-2 and increased the level of Bax combined with Bcl-2. In contrast, a high dose of CORT (30 mg/kg) significantly increased (*p* < 0.05) the level of Bcl-2 combined with Bad and reduced the level of Bax combined with Bcl-2 [one-way ANOVA, F(6, 35) = 107.86, *p* < 0.01 for Bcl-2; F(6, 35) = 96.17, *p* < 0.01 for Bax; F(6, 35) = 136.94, *p* < 0.01 for p-Bad/Bad] (Figure 7).

### The Opposite Effects of Mineralocorticoid Receptor and Glucocorticoid Receptors on Apoptosis Were Mediated by Different Downstream Signaling Pathways

**FIGURE 8:**
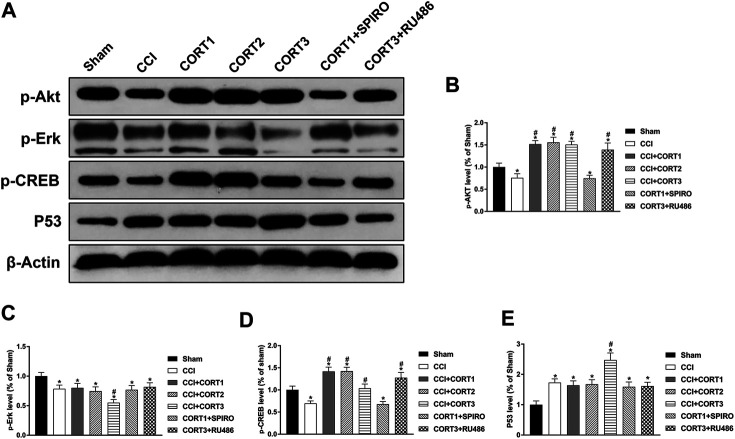
Dose-dependent effects of CORT treatments on apoptosis were mediated by different signaling pathways. **(A)** Representative western blot images showing the levels of *p*-Akt, *p*-Erk, *p*-CREB, and P53. **(B–E)** Quantification of *p*-Akt, *p*-Erk, *p*-CREB, and P53 in the ipsilateral hippocampus on postinjury day 3. The relative band density was measured with ImageJ software (version 1.49) and normalized to that of β-actin, and the concentration percentage compared to that of sham controls was calculated for each sample. *p < 0.05 compared to the sham group; #*p* < 0.05 compared to the CCI control group. The data are presented as the mean ± SD of eight animals in each group.

The PI3K/Akt, MAPK/Erk and P53 pathways are crucial for neural cell survival because they activate CREB and regulate the expression of Bcl-2 protein family members after TBI. In the present study, we tested the levels of p-Akt, p-CREB, p-Erk and P53 (Figure 8A). Our results showed that the levels of p-Akt, p-CREB, and p-Erk in the ipsilateral hippocampus were significantly reduced (*p* < 0.05) (Figures 8B–D), and the level of P53 was significantly increased 3 days after TBI (Figure 8E). Lower doses of CORT (0.3 and 3 mg/kg) significantly increased (*p* < 0.05) p-Akt and p-CREB activation but not p-Erk and P53 activation; however, high-dose CORT significantly inhibited (*p*<0.05) the activation levels of p-Erk, p-CREB and P53, but not that of p-Akt, relative to the levels in the low-dose CORT group [one-way ANOVA, F(6, 55) = 108.698 for p-Akt; F(6, 55) = 99.531 for p-CREB; F(6, 55) = 59.883 for P53; F(6, 55) = 29.990 for *p*-Erk]. The opposite effects caused by different doses of CORT were counteracted by RU486 and SPIRO pretreatment.

## Discussion

We previously found that continuous and full activation of MR induced by low-dose CORT protected hippocampal neural cells from apoptosis in the acute phase after TBI, while overactivation of GR by DEX promoted apoptosis (Zhang et al., 2020a; Zhang et al., 2020b). In the present study, we examined the dual dose-dependent effects of CORT on the MR and GR activation, hippocampal cell survival, rat spatial memory, and receptor downstream signaling pathways after TBI. We found that treatment with low doses of CORT (0.3 and 3 mg/kg) significantly increased MR activation and upregulated Akt/CREB/Bad phosphorylation and Bcl-2 concentration, reduced the number of apoptotic cells, and subsequently improved spatial memory. In contrast, a high dose of CORT (30 mg/kg) exhibited the opposite effect by overactivating GR, upregulating P53 and Bax level, and inhibiting Erk/CREB pathway activation.

Due to their potent anti-inflammatory and immunosuppressive effects, synthetic GCs but not natural GCs have been widely used to treat TBI patients for decades. However, the effectiveness and safety of synthetic GCs in TBI patients have been the focus of discussion and remain controversial (Cohan et al., 2005; Kahles et al., 2013). To date, corticosteroids, particularly synthetic GCs, have still not been recommended for use in TBI patients because short-term use of MP has been found to be harmful to TBI patients (Bratton et al., 2007). However, because of the inadequate understanding of the effects of GC and its two receptors on the CNS, the results of previous clinical studies exhibit obvious limitations. For example, synthetic GCs (for example, MP) with higher affinity for GR than for MR or a GR-specific agonist (DEX) was assessed in most of these studies. Additionally, the GR-induced anti-inflammatory effect of synthetic GCs has been leveraged to reduce brain edema and the increased intracranial pressure after TBI, whereas the GR-mediated neurotoxic effects of synthetic GCs and the MR-mediated neuroprotective effects of natural GCs have been overlooked.

Both our previous experimental studies and other clinical trials revealed the protective effects of natural GCs on TBI (Zhang et al., 2020a, b; Lanterna et al., 2013). Using a rat CCI model, we investigated the corticosteroid receptor mechanism and found that inadequate activation of MR was directly associated with increased neural cell apoptosis in the ipsilateral hippocampus in the acute phase after TBI. Therefore, a proper dose of natural GC replacement might be an effective neuroprotective agent for TBI patients. However, long-term exposure to high levels of endogenous GCs has been reported to be deleterious to the brain. A majority of previous studies (De Kloet et al., 1998; Christian et al., 2011; Sheline et al., 2019) have revealed that chronic increased plasma CORT level induced by chronic stress or long-term CORT treatment impair hippocampal structural integrity and function, including dendritic retraction, reduced neurogenesis in the dentate gyrus, and increased cell loss, which have been associated with increased depressive behaviors and impaired spatial memory. The proper MR/GR activation ratio by slightly increased CORT level induces neuroprotective effects, whereas low or high levels of CORT disrupt the MR/GR balance, leading to neurotoxic effects (Sheline et al., 2019). However, the threshold of GC to maintain the MR/GR balance may significantly vary under distinct pathological conditions and may be affected by the age of the individual treated. Whether short-term administration of high-dose CORT, a mixed agonist of both MR and GR, has deleterious effects on hippocampal cells after TBI is unclear, and the dose at which CORT activates GR and disrupts the MR/GR balance remains unknown.

In the present study, we showed that CORT exhibited dose-dependent effects by activating MR and GR and affecting the MR/GR balance. Low-dose CORT treatment (0.3 mg/kg) fully activated MR in the ipsilateral hippocampus, and the activation levels did not differ in the three CORT treatment groups. Low doses of CORT (0.3 mg/kg and 3 mg/kg) increased the MR activation level and the MR/GR ratio, which might be associated with reduced apoptosis rate in the ipsilateral hippocampus and spatial memory recovery. The GR activation level was significantly increased at a CORT dose of 3 mg/kg and peaked at 30 mg/kg. However, only a high dose of CORT (30 mg/kg) reduced the MR/GR ratio, increased the cell apoptosis rate and aggravated spatial learning impairment. CORT treatment at both the 0.3 mg/kg and 3 mg/kg doses restored the MR/GR ratio and exhibited protective effects. The balance of MR/GR, not the MR or GR alone, determined the dose-dependent effects of CORT, and the protective effects of CORT were counteracted only when the level of GR activation reached a level to reverse the MR/GR ratio.

As a main target of GCs, the hippocampus is one of the most commonly injury structures and is vulnerable to secondary injuries such as apoptosis (Akamatsu et al., 2020). Apoptosis is an important form of cell death contributing form one-third to two-thirds of cell losses after TBI (Zhang et al., 2005). Our previous studies showed that apoptosis mainly occurred in the ipsilateral hippocampus 24 h after TBI and peaked at 48–72 h (Zhang et al., 2020a, b). Bcl-2 family proteins include pro- and antiapoptotic members. The homodimerization of proapoptotic proteins such as Bax can promote the formation of mitochondrial outer membrane permeabilization (MOMP) and the release of cytochrome c from mitochondria, whereas antiapoptotic proteins such as Bcl-2 exert antiapoptotic effects by binding to Bax. Additionally, some BH3-only proteins, such as Bad, can bind to Bcl-2 family members and thus inhibit their antiapoptotic activity (Clark et al., 2001; Piao et al., 2012; Vitale et al., 2018). Therefore, although the expression levels of pro- or antiapoptotic proteins do not necessarily reflect the true apoptosis rate, the Bax/Bcl-2 ratio and Bax and Bcl-2 protein–protein interactions seem to determine whether a cell survives or dies. Although we have reported the MR-mediated antiapoptotic effects of CORT treatment after TBI, the dose-dependent effects of CORT and the downstream mechanisms remain unclear. In the present study, we found that Bax level and the ratio of Bax/Bcl-2 were significantly increased on postinjury day 3. Low doses of CORT (0.3 mg/kg, 3 mg/kg) reduced the Bax/Bcl-2 ratio by upregulating the Bcl-2 level. In contrast, Bax expression and the Bax/Bcl-2 ratio were increased by high-dose CORT treatment (30 mg/kg). In addition, low doses of CORT reduced the level of Bcl-2 binding to Bad and accordingly increased the level of Bax binding to Bcl-2, whereas a high dose of CORT exerted the opposite effects. These data suggested that different doses of CORT exhibited opposite effects on the Bax/Bcl-2 ratio in the hippocampus after TBI. The dose-dependent effects of CORT were mediated by two receptor systems, MR and GR. However, their target proteins were different; MR increased Bcl-2 level, whereas GR upregulated Bax level.

Several intracellular signaling pathways were previously found to be involved in neuronal apoptosis after TBI (Noshita et al., 2002; Farook et al., 2013; Kowalski et al., 2019), such as the Akt pathway, the activation of which exerts antiapoptotic effects through the inactivation of Bad and phosphorylation of CREB (Zhang et al., 2006). As a key transcription factor, CREB plays important roles in neuronal survival and neurogenesis in many conditions by regulating the expression of genes, including those encoding brain-derived neurotrophic factor (BDNF) and Bcl-2 (Titus et al., 2013; Pardo et al., 2016). In addition, crosstalk between components of apoptosis-related pathways is very common. For instance, CREB has been reported to be activated by several signaling pathways after TBI, including the IP3K/Akt, cyclic AMP-dependent protein kinase/protein kinase A, and the MAPK/Erk pathways (Su et al., 2017; Schreiber et al., 2020; Sadeghi et al., 2021). However, the effect of a given signaling pathway on CREB activation remains controversial because the overall CREB activation level is generally determined by interactions of multiple signaling pathways, not a single pathway. In addition, even the same protein can be differentially activated depending on its location in the brain and time after TBI.

Previous studies have found that chronic stress or prolonged natural GC administration aggravates neuronal apoptosis by suppressing CREB activation (Licznerski et al., 2018) and that transient GC elevation exhibits neuroprotective effects by upregulating CREB activation and BDNF expression (Numakawa et al., 2013; Arango-Lievano et al., 2015). However, the receptor mechanism that contributes to these contradictory results remains unclear, and the dose-dependent effects of CORT on CREB need to be studied further. In the present study, we showed that Erk, Akt and CREB activities were reduced on postinjury day 3 when the cell apoptosis rate peaked in the ipsilateral hippocampus. Lower doses of CORT (0.3 mg/kg and 3 mg/kg) upregulated the activation levels of *p*-Akt and *p*-CREB. However, when the dose was increased to 30 mg/kg, short-term use of CORT led to the opposite effects: inhibition of Erk and CREB activity and upregulation of P53 and Bax level. The GR-specific inhibitor RU486 and MR-specific inhibitor SPIRO counteracted the opposite effects induced by high- and low-dose CORT treatment on the abovementioned proteins, which proved that the proapoptotic effect of GR and the antiapoptotic effect of MR were mediated by different downstream signaling pathways.

The limitations of the present study are as follows: (1) We used specific antagonists of GR and MR (RU486 and spironolactone) to inhibit the activation of GR and MR; however, control groups, namely, the sham + SPIRO, sham + RU486, CCI + SPIRO, and CCI + RU486 groups, were not treated with these antagonists; therefore, the intrinsic effects of each antagonist on stress activity, inflammation, and cell survival before and after TBI were not evaluated. (2) In the present study, H&E staining, which can effectively show cell morphological changes after TBI, such as changes characteristic of cell necrosis and apoptosis, including cell shrinkage, chromatin condensation, DNA fragmentation and the formation of apoptotic bodies, was evaluated to quantify the surviving neurons in subareas of the hippocampus (CA1, CA2, CA3, and DG). Although most of the cells in these areas are hippocampal neurons, a small percentage comprise glial cells. Neuron-specific marker immunostaining, such as NEUN staining, would have been provided more accurate neuron counts. (3) We used the sham control group to examine brain injury after CCI because the rat brains in this group were not influenced by craniotomy, but we did not examine the effects of craniotomy on brain injury by including a naïve group. (4) We used only male rats to exclude the effects of sex and sex hormones on glucocorticoids and their receptors. Therefore, in subsequent studies, we will directly investigate the GR- and MR-induced dose-dependent effects of CORT using GR- or MR-specific knockout mice and examine sex-dependent MR-induced neuroprotective effects.

In conclusion, our study showed the dose-dependent effects of short-term CORT treatments on spatial memory, cell apoptosis in the ipsilateral hippocampus, and possible receptor downstream signaling pathways after TBI. Our results suggest that low doses of endogenous GC maintain a proper MR/GR activation ratio and are thus beneficial and may be recommended for treating patients with TBI. However, although the safe dose range of CORT is very wide (0.3–3 mg/kg), there is a point at which CORT treatment ceases to induce antiapoptotic effects and begins to promote proapoptotic effects. A much higher dose (30 mg/kg) can also lead to neurotoxic effects, even with short-term use.

## Data Availability

The datasets used and/or analyzed for the current study are available from the corresponding author on reasonable request. Requests to access the datasets should be directed to BL, liubaiyun12121963@163.com.
